# The Role of Toll-Like Receptors in Colorectal Cancer Progression: Evidence for Epithelial to Leucocytic Transition

**DOI:** 10.3389/fimmu.2014.00429

**Published:** 2014-10-20

**Authors:** Kimberly A. Luddy, Mark Robertson-Tessi, Narges K. Tafreshi, Hatem Soliman, David L. Morse

**Affiliations:** ^1^Department of Cancer Imaging and Metabolism, Imaging and Technology Center of Excellence, H. Lee Moffitt Cancer Center, Tampa, FL, USA; ^2^Department of Integrated Mathematical Oncology, H. Lee Moffitt Cancer Center, Tampa, FL, USA; ^3^Don and Erika Wallace Comprehensive Breast Program, Center for Women’s Oncology, H. Lee Moffitt Cancer Center and Research Institute, Tampa, FL, USA

**Keywords:** colorectal cancer, toll-like receptors, epithelial to leucocytic transition, ELT, metastasis, immune evasion, cell plasticity, inflammation

## Abstract

Toll-like receptors (TLRs) are expressed by immune cells, intestinal epithelium, and tumor cells. In the homeostatic setting, they help to regulate control over invading pathogens and maintain the epithelial lining of the large and small intestines. Aberrant expression of certain TLRs by tumor cells can induce growth inhibition while others contribute to tumorigenesis and progression. Activation of these TLRs can induce inflammation, tumor cell proliferation, immune evasion, local invasion, and distant metastasis. These TLR-influenced behaviors have similarities with properties observed in leukocytes, suggesting that tumors may be hijacking immune programs to become more aggressive. The concept of epithelial to leucocytic-transition (ELT) is proposed, akin to epithelial to mesenchymal transition, in which tumors develop the ability to activate leucocytic traits otherwise inaccessible to epithelial cells. Understanding the mechanisms of ELT could lead to novel therapeutic strategies for inhibiting tumor metastasis.

## Introduction

Toll-like receptors (TLRs) are a diverse family of pattern recognition receptors expressed by immune cells from both the innate and adaptive arms of the immune system (1–4). Hence, TLRs stimulate both innate and adaptive immune responses to invading pathogen-associated molecular patterns (PAMPs) as well as danger-associated molecular patterns (DAMPs) from damaged epithelial cells. Ligand specificity, downstream signaling, and subsequent immune stimulation vary greatly among the TLR subtypes (5, 6). The complex expression patterns of TLRs differ among cell types, physiological location, and are controlled by the microenvironment (7–9). In the healthy gastrointestinal (GI) tract, TLRs are expressed by intestinal epithelial cells (IEC) and play a role in immune modulation and tissue homeostasis by stimulating the immune response to bacterial pathogens, attenuating the immune response against favorable microbes, sensing breakdown of the protective intestinal barriers, and triggering proliferative signaling (6, 10, 11). The lumen of the gut is subjected to continual interactions with the microbiome and would be in a constant state of inflammation were it not for the controlled expression and normal function of these TLRs (2, 6).

Inflammation has been implicated as an underlying factor in tumorigenesis and cancer progression, leading transformed cells to develop the “hallmarks of cancer” (12, 13). Some of the same TLRs (TLR 2–4) that normally regulate inflammation in the gut are also found to be aberrantly expressed in colorectal cancers (CRCs) (14, 15). Overexpression of TLR4 in CRC is associated with poor survival (16). Deletion of TLR4, its signaling partner Myd88, or absence of its ligand LPS in the colon can lead to increased or reduced inflammation, depending on the cancer subtype, which can then lead to either increased or decreased tumorigenesis and tumor progression (17, 18). It is notable that the role of TLRs in CRC reflects a “natural history” of selection events that lead from normal TLR function in unaffected colon tissue and throughout all stages of CRC progression. More specifically, the normal role of TLR immune modulation in the gut involves the controlled release of cytokines and danger signals that stimulate the immune response to bacterial pathogens and attenuate the immune response against favorable microbes (6, 10). Microbial imbalance and/or dysregulation of these responses leads to chronic inflammation of the bowel (15). Chronic inflammation is correlated with initiation of cancer development and in the progression of cancer into more aggressive forms of malignancies (2). Aberrant TLR signaling and the resulting cytokine imbalance leads to increased epithelial proliferation and decreased cell death (19). Additionally, an active immune environment creates selection pressures for initiating cancer cells resulting in the evolution of an immune-evasive tumor phenotype (14). Furthermore, TLR dysregulation is implicated in cancer invasion and metastasis (2, 19–21). Understanding the role of TLRs in the natural evolution of metastatic disease is crucial for developing new therapies and optimizing current treatments.

## Role of TLRs in Inflammation-Mediated Tumorigenesis

The intestines house approximately 70% of the body’s immune cells under normal conditions (10). Signaling between these immune cells, commensal bacteria, and IECs is critical for normal digestion and protection against invading pathogens (6). TLRs are key modulators of the immune system of the GI tract. In order to maintain homeostasis and suppress immune responses to commensal bacteria (11), TLR expression and signaling are tightly controlled in this environment (6, 20). However, these controls are disrupted in diseases such as Crohn’s disease and ulcerative colitis, resulting in chronic inflammation (11, 15). Inflammation is linked to cancer through two pathways: extrinsic inflammation induced by non-transformed cells (e.g., invading pathogens or autoimmune disease), and intrinsic inflammation induced by transformed cells (22). In CRC, TLRs are involved in both. Autoimmune diseases cause chronic, smoldering levels of inflammation that predispose individuals into developing CRC (22). Once initiated, tumors can intrinsically activate inflammation through TLR binding by cancer-related DAMPs. Intrinsically and extrinsically induced TLR activation results in tumor-promoting inflammation through NF-κB signaling, leading to expression of the inflammatory cytokines IL-1β, TNFα, and IL-6 (17). This aberrant expression by tumor cells in early carcinogenesis can recruit tumor-promoting immune cells, leading to inflammation and protection from cytotoxic immune cells. Additional data from Kim et al. links mutations in p53 and PTEN to SOCS-mediated activation of IL-6 signaling, leading to intrinsic inflammation (23). Since p53 mutation is a very common event in the natural history of CRC, this is likely a major mechanism of tumor-induced inflammation. Additionally, inflammation can drive genetic and epigenetic changes in cells as well as possible alterations in lineage differentiation programs leading to increased plasticity. This process is also thought to involve NF-κB signaling; however, further studies are needed (22, 24).

## Role of TLRs in inflammation-Mediated Proliferation and Survival

Inflammatory pathways are tightly linked to aberrant proliferation and resistance to cell death, which are key cancer hallmarks that can be mediated through TLR activation (14). IECs are the barrier layer that protects the interstitial layers from the changing exterior environment of the GI tract. Infiltrating bacteria and the resulting immune response can cause tissue damage. To prepare for this, IECs utilize TLR4 signaling as an early warning system to initiate proliferation, maintain tissue integrity, and protect the interstitial compartments (6). Tumors can co-opt this system, allowing cells to proliferate unchecked (25).

Tumor growth is further fueled through an overabundance of growth factors (e.g., TGF-β, IL-8, CXCR4, and VEGF) (15), a decline in immune surveillance, and the evolution of mobile and invasive phenotypes. TLR expression on tumor cells stimulates the release of cytokines that recruit favorable immune cells further driving proliferation. Additionally, the release of cytokines and chemokines due to TLR signaling generates an autocrine loop that further stimulates tumor cell growth. The cumulative result is tumor control over its own environment.

## Role of TLRs in Immune Evasion

An active immune environment selects for the natural evolution of cancer cells with decreased immunogenic phenotypes. TLR expression in tumors can confer the advantages of both immune evasion and immunosuppression (26). Often pro-inflammatory signals reduce elements of the adaptive immune response. TLR signaling causes a shift in this response from anti-tumor to pro-tumor by affecting the balance toward inflammation and suppression of anti-tumor immunity. Direct TLR activation results in production of immunosuppressive cytokines IL-10 and TGF-β (14, 27), as well as increased expression of immune modulating surface markers PD-L1 and HLA-G (19, 20, 28). These secreted and surface proteins have a tolerizing effect on immune cells. TLR-activated IECs induce the transformation of dendritic cells (DC) into an antigen-specific CD103+ phenotype. These DC promote contact-dependent antigen-specific regulatory T cells (Tregs) that express gut-homing integrins, which further attenuates the anti-tumor immune response (10). Each of these mechanisms are used in the healthy gut to avoid food hypersensitivity or auto-immune diseases. However, dysregulation through abnormal TLR expression can lead to malignant progression.

## Role of TLRs in Invasion and Metastasis

The most dangerous effect of tumoral TLR signaling is the acquisition of invasive and metastatic tumor phenotypes (29). Ninety percent of patients who succumb to their disease have metastatic lesions (30). TLR expression in tumors is linked to increased grade and distant metastasis (2, 18, 21, 31). The ability of a tumor cell to detach from its epithelial neighbors, break through the basement membrane, and invade nearby tissues is, in part, the result of a long history of aberrant TLR signaling. In CRC, TLR-mediated alterations of the immune system components in the tumor microenvironment can change intracellular signaling (NF-κB), integrin expression (B1 integrin), and motility (29, 32). Activation of TLR4 by LPS *in vitro* and *in vivo* induces epithelial to mesenchymal transition (EMT) and invasive phenotypes in certain cell lines (29, 33).

Immune cells are educated by tumor-secreted factors and then actively migrate through the lymphatic vessels and secondary lymphoid organs. These tightly gated organs allow entry and passage to soluble antigens and select immune cell phenotypes, and yet lymph nodes are often the first site of metastasis (34). While it was once thought that tumors cells passively filter into draining lymph nodes, it has recently been shown that tumor cells require chemokine-mediated (CCR7 and CCR8) active transport through the subcapsular sinus epithelium (35, 36). Furthermore, it has been shown that tumor-mediated lymphatic remodeling of peritumoral lymph vessels and draining lymph nodes facilitates metastasis (37–40). TLRs may play a role in this metastatic process, since TLR activation leads to increased expression of CCR7 and CCR8 (41), which are key molecules expressed by leukocytes to access lymphatics (35, 42). This suggests that the tumor cells can harness existing leucocytic mechanisms to begin the metastatic cascade through the lymph nodes.

Lymphocytes typically traffic throughout the body to sites of inflammation, using chemokines, selectins, and integrins as homing signals (43). Many metastatic tumors have been shown to use the expression of these same molecules to colonize distal sites (44, 45). As an example, CXCR4 is a well-characterized bone marrow homing receptor expressed by T cells (46); research has found that both prostate cancers (47) and breast cancers (48) that metastasize to the bone commonly express CXCR4. CRC typically metastasizes to the liver or lung. Aberrant expression of CXCR3, CXCR4/CXCR7, and CCR6 are commonly found in liver and lung metastasis of colon cancer (49–55). Ligands for these receptors (CXCL19, SDF-1, and CCL20, respectively) are highly expressed in the liver and lungs of metastatic CRC patients (53, 56–58). Local inflammation in these organs induces ligand expression and preferential organ metastasis is determined by their expression (59, 60).

Alteration in integrin signaling is another metastatic mechanism induced by TLR signaling (26). Integrin signaling is used in healthy systems to aid immune cell trafficking (61). Aberrant expression of these integrins via TLR signaling allows circulating tumor cells to respond to the same trafficking mechanisms that an immune cell uses to migrate to distal sites (2, 32, 62, 63). Similar examples have been shown with integrins in colon cancer (64), breast cancer (65), and melanoma (66). These expressed surface markers are a natural part of the lymphocytic trafficking system, and their expression on tumor cells could be evidence that tumor cells use leucocytic trafficking mechanisms to metastasize.

## Epithelial to Leucocytic Transition

The co-opting of immune cell signaling and migration mechanisms by tumor cells is well documented, with many citing the plasticity of tumor cells and inappropriate gene expression as the underlying cause of treatment resistance and metastatic growth (13, 67–70). Pressures from cytotoxic immune cells, abundant inflammation, cytotoxic drugs, and targeted therapies push tumor cells into plastic states where they may begin to access programed mechanisms outside of their usual function (68). The survivors of these selection pressures are adaptive and dynamic cells, many of which express patterns of proteins found in other normal cell types (70, 71). These protein expression patterns have been used to define and detect EMT, for example. An increasing number of publications suggest that although EMT is important in locally invasive disease, it is not enough to allow tumor cells access to lymph nodes, lymphatic and vascular systems, as well as entrance and settlement into distant tissues (35, 69, 72, 73). Others hypothesize a myeloid lineage expression pattern gained from horizontal gene transfer and Lamarckian inheritance, tumor cell myeloid cell fusion, or a possible myeloid cell origin (69, 74–76). Here, we build on these observations and propose a new concept, the transition from epithelial phenotype to leucocytic phenotype.

Immune cells of myeloid and lymphoid origins house a diverse set of mechanisms that make them perfect trafficking cells. They can shift their metabolism easily, survive in low oxygenated areas, roll along the endothelium in the presence of high shear forces, read integrin codes, and facilitate tissue specific extravasation (77–79). As described above, the aberrant expression of TLRs by CRC cells results in the acquisition of a number of tumor-promoting mechanisms. At the same time, these mechanisms are key properties of the immune system, as is TLR expression. In a broad sense, immunosuppression, migration through tissue, intra- and extravasation through lymph and blood vessels, rapid proliferation, altered metabolism, and homing to specific tissues are key hallmarks of both cancer and the immune system.

**Figure 1:**
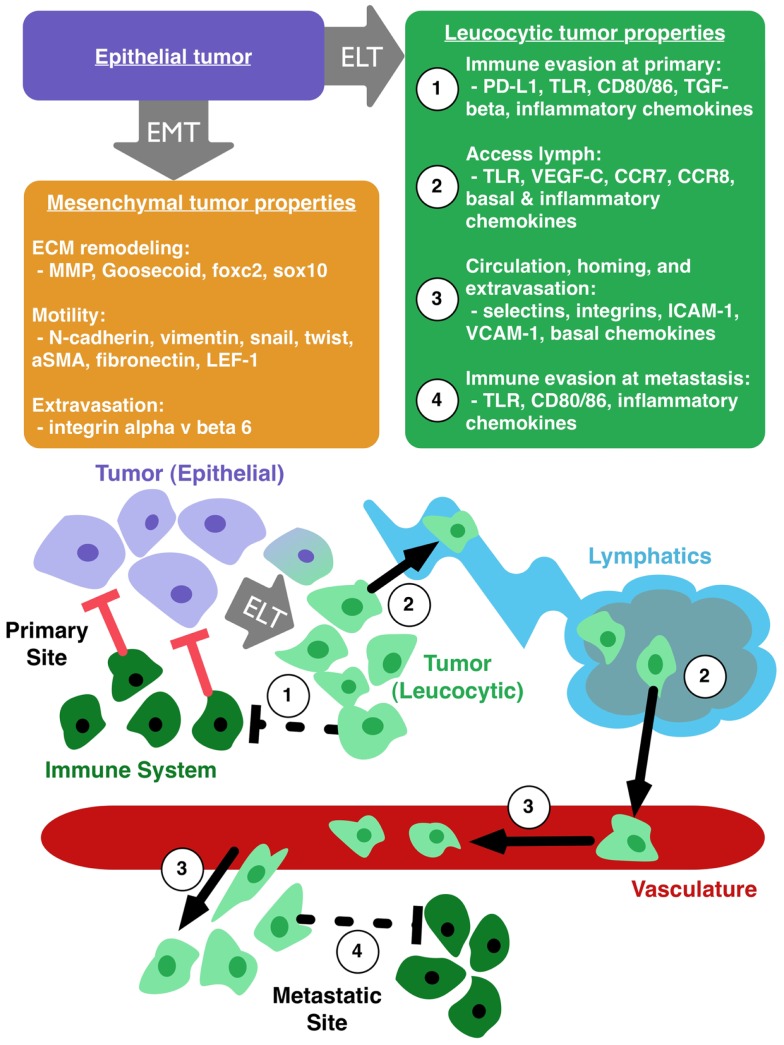
**Epithelial to leucocytic transition (ELT) is the acquisition of immune properties by tumor cells**. Epithelial tumor cells (purple box) can make transition to a mesenchymal phenotype (orange box), which enhances local motility and remodeling of the extracellular matrix (ECM). Tumor cells undergoing ELT (green box) can gain the ability to (1) evade the immune system at the primary tumor site, (2) access the lymphatic system, (3) circulate through the vasculature, home to favorable sites of metastasis, and extravasate into a metastatic niche, and (4) avoid destruction by the immune system at the site of metastasis.

Pathogenic EMT has its roots in normal embryogenesis. In cancer, this transition results in epithelial cells with a range of mesenchymal protein expression. These alterations increase motility and invasive capability of tumor cells, but do not necessarily explain immunoevasion, lymphatic access, and metastatic spread (35, 69, 72). We therefore propose the parallel concept of epithelial to leucocytic transition (ELT) as a framework, akin to EMT, with which to understand the metastatic properties of cancer cells. Figure 1 illustrates the primary properties gained by tumor cells that undergo ELT. We consider ELT to be a partial transition in which epithelial cells retain their epithelial origin while at the same time acquiring a set of leucocytic traits. Tumor cells co-opt many mechanisms of the immune system for their own transport and these mechanisms are activated by proteins typically reserved for the immune response. A leucocytic tumor cell expresses proteins that allow for regulation and co-opting of the immune system such as PD-L1, CD80/86, TLR, TGF-β, CCL4, and CCL5 (80) (Figure 1, properties 1 and 4). Additional leucocytic proteins (CXCR4, CCR7, CCR8) facilitate invasion and proliferation within lymph nodes (Figure 1, property 2) (35, 42, 81). Processes critical to survival in circulation, homing to tissue specific sites, and successful extravasation are mediated by E/P-selectins, L-selectin ligands, α4β1, ICAM-1, and VCAM-1 (61, 73, 82–86) (Figure 1, property 3). By harnessing mechanisms usually reserved for immune cells, tumor cells gain the ability to become more aggressive. In the case of TLRs, a cycle of overexpression and resulting inflammation promotes plasticity of the epithelial phenotype. This plasticity permits tumor cells to undergo ELT, accessing immune programs that facilitate invasion and metastasis of the cancer. ELT, as with other plastic states, is likely transient, making the evaluation of these phenotypes a significant challenge. TLR-mediated evolution of CRC may be a good model to study how ELT occurs, since TLRs are primarily seen in immune cells and the overexpression of TLRs appears to promote an immune-like phenotype in CRC.

Understanding the acquisition of the leucocytic phenotype could reveal key targets that would prevent CRC cells from accessing dangerous invasion and trafficking mechanisms through a plastic transition. Simply antagonizing TLRs and associated molecules may not be enough, since resistance is likely to develop. However, if the mechanisms of plasticity induced by TLRs are understood, new targets may be developed to inhibit ELT.

It is important to note that the key functional activities of immune cells, specifically the CD8 cytotoxic T-cell phenotype and the antibody producing activated B-cell phenotypes, have not yet been described in tumor cells. However, other cytotoxic mechanisms utilized by immune cells have been seen in normal and neoplastic epithelial cells. Tumor cell cannibalism, resembling phagocytosis, of neighboring apoptotic cells as well as infiltrating immune cells has been seen during times of metabolic stress (87). During mammary involution, epithelial-derived FAS plays a role in FASL-mediated cell death (88). Tumor cells can secrete FAS, TNFa, and TGFb, proteins capable of promoting and inhibiting epithelial cell death (89–91). Additionally, PD-L1 proteins on tumor cells result in T-cell anergy and apoptosis (92, 93). Although, none of these represent the antigen-specific killing of the adaptive immune system, it is our opinion that further exploration is needed to determine how far epithelial cells can evolve to obtain immune-like processes and that cell killing can not yet be included or excluded from that hypothesis.

## Conclusion

Originally, lymphatic dissemination into draining lymph nodes was considered a clear indicator of prognosis and was attributed to tumor chronology based on the correlation of tumor volumes and lymph node metastasis. However, later larger studies often showed conflicting results. Jatoi et al. (94, 95) and others attributed these differences to the tumor phenotype as opposed to a simple passage of time. This means that tumor phenotypes can exist on a continuum from slow growing with late lymph node metastasis to aggressive early disseminators much more capable of exiting the lymph node and establishment at distant sites (94, 95). While lymph node positivity is a useful tool for treatment decisions understanding the complexities of these aggressive phenotypes is key to halting the lymphatic dissemination of cancer.

Many host parameters contribute to natural progression of tumor metastasis and the extent of tumor cell plasticity is not yet fully appreciated. In an opinion article on tumor and immune cell plasticity, Holzel et al. (68) recognize the similarity between cancer cells and immune cells by linking inflammation and evolutionary pressures to the creation of plastic phenotypes. We think that this idea needs to be taken further to include a plastic transition to an immune-like phenotype, i.e., ELT, in the context tumor development, invasion, metastasis, and resistance to therapies. Specifically in CRC, the tumor cells acquire many hallmarks of the immune system, and this transition is intimately tied to aberrant TLR expression. By considering TLR expression in the context of ELT, the transition to a migratory immune-like and therefore metastatic phenotype might be better understood, and therefore, lead to better therapeutic strategies.

## Conflict of Interest Statement

The authors declare that the research was conducted in the absence of any commercial or financial relationships that could be construed as a potential conflict of interest.
